# Clinical, paraclinical and serological findings in Susac syndrome: an international multicenter study

**DOI:** 10.1186/1742-2094-11-46

**Published:** 2014-03-08

**Authors:** Sven Jarius, Ilka Kleffner, Jan M Dörr, Jaume Sastre-Garriga, Zsolt Illes, Eric Eggenberger, Colin Chalk, Marius Ringelstein, Orhan Aktas, Xavier Montalban, Kai Fechner, Winfried Stöcker, Erich B Ringelstein, Friedemann Paul, Brigitte Wildemann

**Affiliations:** 1 Molecular Neuroimmunology, Department of Neurology, University of Heidelberg, Heidelberg, Germany; 2 Department of Neurology, University of Heidelberg, Münster, Germany; 3 NeuroCure Clinical Research Center and Clinical and Experimental Multiple Sclerosis Research Center, Department of Neurology, Charité-Universitätsmedizin, Berlin, Germany; 4 Servei de Neurologia/Neuroimmunologia, Centre d’Esclerosi Múltiple de Catalunya (Cemcat), Hospital Universitari Vall d’Hebron (HUVH), Barcelona, Spain; 5 Department of Neurology, Odense University Hospital, Institute of Clinical Research, University of Southern Denmark, Odense, Denmark; 6 Department of Neurology and Ophthalmology, Michigan State University, East Lansing, MI, USA; 7 Department of Neurology and Neurosurgery, McGill University, Montreal, Canada; 8 Department of Neurology, University of Düsseldorf, Düsseldorf, Germany; 9 Institute for Experimental Immunology, affiliated to Euroimmun, Luebeck, Germany

**Keywords:** Susac syndrome, Susac’s syndrome, anti-endothelial cell antibodies, AECA, indirect immunofluorescence, laboratory test, encephalopathy, hearing loss, visual impairment, branch retinal artery occlusion, BRAO

## Abstract

**Background:**

Susac syndrome (SuS) is a rare disorder thought to be caused by autoimmune-mediated occlusions of microvessels in the brain, retina and inner ear leading to central nervous system (CNS) dysfunction, visual disturbances due to branch retinal artery occlusions (BRAO), and hearing deficits. Recently, a role for anti-endothelial cell antibodies (AECA) in SuS has been proposed.

**Objectives:**

To report the clinical and paraclinical findings in the largest single series of patients so far and to investigate the frequency, titers, and clinical relevance of AECA in SuS.

**Patients and methods:**

A total of 107 serum samples from 20 patients with definite SuS, 5 with abortive forms of SuS (all with BRAO), and 70 controls were tested for AECA by immunohistochemistry employing primate brain tissue sections.

**Results:**

IgG-AECA >1:100 were detected in 25% (5/20) of patients with definite SuS and in 4.3% (3/70) of the controls. Median titers were significantly higher in SuS (1:3200, range 1:100 to 1:17500) than in controls (1:100, range 1:10 to 1:320); IgG-AECA titers >1:320 were exclusively present in patients with SuS; three controls had very low titers (1:10). Follow-up samples (n = 4) from a seropositive SuS patient obtained over a period of 29 months remained positive at high titers. In all seropositive cases, AECA belonged to the complement-activating IgG1 subclass. All but one of the IgG-AECA-positive samples were positive also for IgA-AECA and 45% for IgM-AECA. SuS took a severe and relapsing course in most patients and was associated with bilateral visual and hearing impairment, a broad panel of neurological and neuropsychological symptoms, and brain atrophy in the majority of cases. Seropositive and seronegative patients did not differ with regard to any of the clinical or paraclinical parameters analyzed.

**Conclusions:**

SuS took a severe and protracted course in the present cohort, resulting in significant impairment. Our finding of high-titer IgG1 and IgM AECA in some patients suggest that humoral autoimmunity targeting the microvasculature may play a role in the pathogenesis of SuS, at least in a subset of patients. Further studies are warranted to define the exact target structures of AECA in SuS.

## Introduction

Susac syndrome (SuS) is a rare disorder that is thought to be caused by autoimmune-mediated occlusions of microvessels in the brain, retina and inner ear [1] which lead to a characteristic clinical triad of central nervous system (CNS) dysfunction, visual disturbances and hearing deficits [1-5]. Typical findings in patients with SuS include branch retinal artery occlusions (BRAO) detectable on retinal fluorescein angiography, characteristic callosal lesions on cranial MRI, and evidence of sensorineural hearing loss [1,6]. The three index events defining SuS may occur simultaneously or, more often, successively. The disease may be monophasic or may follow a relapsing or a chronic-progressive course.

The exact prevalence of SuS is unknown but is considered to be low; since the syndrome was first described in 1973 only about 300 patients have been reported worldwide [1]. Accordingly, most of our current knowledge on SuS is based on case reports or small case series, only very few of which have included more than four patients [1].

Although spontaneous recovery and long-term remission have been described, many patients respond to immunosuppressive agents, suggesting a possible autoimmune pathogenesis [1,5,7]. A role for anti-endothelial cell antibodies (AECA) in the pathogenesis of SuS was proposed by Susac and co-workers in two review articles in 2007 with reference to unpublished data [7,8]. However, no original studies had been published by then to substantiate this claim. When examining the only serum samples from a patient with SuS regularly seen at our center at that time, we indeed found evidence of an anti-endothelial humoral immune response [9], in keeping with those anecdotal reports. However, it remained unknown whether AECA are a common phenomenon in SuS or whether that was an isolated finding. A more recent study seems to confirm the presence of AECA in SuS, but its results are challenged by a number of methodological issues [10].

Here, we provide a summary of the most important clinical and paraclinical findings associated with SuS from one of the largest single cohorts of patients with SuS studied to date. In addition, we present data on the frequency, titers and clinical relevance of AECA in SuS.

## Patients and methods

Twenty-five patients with SuS and 70 controls, comprising 25 patients with multiple sclerosis (MS) (relapsing remitting MS in 18, secondary progressive MS in 5, and primary progressive MS in 2), 25 patients with connective tissue disorders (CTD) with CNS involvement (systemic lupus erythematosus (SLE) in 17, Sjögren syndrome (SS) in 1, SLE and secondary SS in 1, Sharp syndrome in 5, and scleroderma in 1) and 20 healthy controls (HC), were analyzed for serum AECAs using a tissue-based indirect immunofluorescence assay (IFA) [11]. Patients with SuS were further stratified according to clinical, fluorescence angiographic and MRI findings: While all patients in group I (n = 20) had encephalopathy, hearing loss and visual disturbances (‘complete SuS’), group II consisted of patients (n = 5) in whom only two of the three typical sites (brain, retina, inner ear) were affected but in whom a diagnosis of SuS was likely due to the presence of BRAO (‘limited SuS’). In addition, 12 follow-up samples from 7 patients with definite SuS were available, adding up to a total number of 107 samples tested, including 37 from patients with SuS.

All patients and controls were of Caucasian origin (countries of origin are given in Table 1). The sex ratio (m:f) was 1:2.6 in the SuS group (1:3 in the definite SuS subgroup) and 1:3.4 in the control group (*P* = n.s.). The median age at blood sampling was 32 years in the SuS group and 35 years in the control group (*P* = n.s.) (Table 1).

**Table 1:** Epidemiological data from the total cohort and disease and control subgroups

	**Subjects**^**a**^	**Samples**	**Median age (range)**		**Sex (m:f)**	
All	90	90	33 (16 to 79)		1:3.1	
**SuS**	25	37	32 (19 to 62)	*P* = n.s.^b,d^	1:2.6	*P* = n.s.^c,d^
Definitive SuS	20	32	32 (20 to 62)		1:3	
Limited SuS	5	5	30 (19 to 44)		1:1.5	
**Controls**	70	70	35 (16 to 79)		1:3.4	
HC	20	20	28 (24 to 50)		1:3	
MS	25	25	35 (21 to 67)		1:2.6	
CTD	25	25	44 (16 to 79)		1:5.3	

CTD, connective tissue disorders; HC, healthy controls; MS, multiple sclerosis; n.s., not significant; SuS, Susac syndrome. ^a^All subjects were of Caucasian origin (SuS: Germany (n = 20), Hungary (n = 2), Spain (n = 1), Canada (n = 1), USA (n = 1); controls: Germany). ^b^Mann Whitney *U* test. ^c^Fisher’s exact test. ^d^Total SuS group vs controls and definite SuS subgroup vs controls.

All serum samples were stored at −80°C until testing. The median time between SuS onset and blood sampling was 5 years (range 0 to 18), and the median time between the most recent SuS attack and blood sampling was 39.5 months (range 0 to 165). While 20 samples were taken from untreated patients, 15 were taken during periods of active immunomodulatory or immunosuppressive treatment; in 2 cases the exact treatment status at the time of blood sampling was unknown. A total of 35.1% (13/37) of samples were obtained during phases of active disease. All patients with SuS had been treated at some point in time, most commonly with platelet aggregation inhibitors or anticoagulants (acetylsalicylic acid in 17, clopidogrel in 1, dipyridamole in 1, and fondaparinux in 1). Five patients were treated with nimodipine and 1 with losartan. Immunomodulatory (IM) and immunosuppressive (IS) treatments used at some point in time included steroids in 18 patients, cyclophosphamide in 10, intravenous immunoglobulins in 9, mycophenolate mofetil in 6, azathioprine in 4, and methotrexate in 2; overall, 23/25 patients (92%) had received IM and/or IS treatments at least once.

Antibodies to CNS tissue were analyzed by indirect immunofluorescence on adult primate (*Macaca mulatta*), rat, and mouse cerebellum cryosections (Euroimmun, Luebeck, Germany) as described [9,11,12]. Briefly, unfixed or fixed cryosections (10% formalin for 4 min and 3-[(3-cholamidopropyl) dimethylammonio]-1-propanesulfonate (CHAPS) 1% in PBS for another 4 min), respectively, were blocked with 10% goat serum and subsequently incubated with patient serum for 1 h. Human IgG, IgM and IgA binding to CNS tissue were detected by the use of prediluted polyclonal goat anti-human IgG, anti-human IgM, or anti-human IgA, respectively, conjugated to fluorescein isothiocyanate (FITC) (Euroimmun). Sections were mounted using ProLongGold mounting medium (Invitrogen, Darmstadt, Germany) containing 4′,6-diamidino-2-phenylindole (DAPI; 1:1000). Each incubation step was followed by three washes in PBS. For evaluation of IgG subclasses, serum samples were tested by immunohistochemistry (IHC) on mouse cerebellum sections as described above, with the following modifications: unconjugated sheep anti-human IgG antibodies specific for IgG subclasses (Binding Site, Germany) were substituted for the FITC-labeled goat anti-human IgG antibody, and Alexa Fluor (AF) 568-labelled donkey anti-sheep IgG (Invitrogen; absorbed against primate IgG) was used to detect the subclass-specific antibodies. In addition, all AECA-positive samples were tested for neuromyelitis optica (NMO)-IgG/aquaporin4 (AQP4) antibodies, the staining pattern of which shows similarities with that of AECA when tested by IHC, using a highly sensitive and specific commercial cell-based assay employing recombinant human AQP4 as previously described [13-15]. In addition, double staining of monkey cerebellum sections with a commercial antibody to AQP4, detected using AF568-labelled anti-rabbit IgG, and sera of AECA positive patients, detected using FITC-labeled, anti-human IgG was performed as previously described [12].

All data were analyzed in an anonymized fashion as required by the institutional review board of the University of Heidelberg. The Mann–Whitney *U*-test (2-tailed) was used to test for significant differences between continuous variables, and Fisher’s exact test (2-tailed) to compare proportions. All tests should be understood as constituting exploratory data analysis, such that no adjustments for multiple testing have been made. Microsoft Excel 2003 and GraphPad Prism 4 were used for statistical analyses.

## Results

### Clinical and paraclinical findings

#### Disease onset

The median age at SuS onset was 28 years (range 17 to 56). Signs and symptoms at disease onset included hypoacusis in 5 patients (20%), visual disturbances in 6 (24%), and/or signs and symptoms of encephalopathy other than headache in 18 (72%); in none of the SuS patients were all three predilection sites simultaneously affected at disease onset. Fifteen patients (60%) had only encephalopathic but no visual or auditory symptoms at onset.

#### Time to diagnosis

The time between disease onset and correct diagnosis of SuS ranged between 0 to 126 months (median 7 months). Diagnoses suggested at first presentation by the then treating physicians included MS (5×), ‘(chronic) inflammatory autoimmune disorder’ (3×), ‘autoimmune demyelinating disorder’ (1×), ‘encephalitis’ (4×), ‘parainfectious focal encephalitis’ (1×), ‘encephalopathy’ (2×), ‘vasculitis’ (2×), ‘possibly systemic lupus erythematosus’ (2×), ‘sudden acute hearing loss’ (2×), ‘possible morbus Menière’ (1×), ‘possible Cogan syndrome’ (1×), and ‘peripheral vertigo’ (1×).

#### Disease course

At the most recent follow-up (median disease duration 4.5 years, range 0 to 17), the disease had taken a relapsing course in 19 patients (76%; median disease duration 4 years) and was monophasic in 6 (median disease duration 4 years). The median number of attacks at last follow-up was 3 (range 1 to 6).

#### Outcome

Neuropsychological symptoms, as reported by the treating physicians, were present at last follow-up in 16/25 patients (64%) and included ‘fatigue’, ‘mild psycho-organic syndrome’, ‘disorientation’, ‘mnestic deficits’, mild to extremely severe ‘cognitive impairment’, and ‘encephalopathy’. Motor symptoms were present in 6/25 patients (24%) and included ‘spasticity’, ‘pyramidal signs’, ‘paresis’, ‘spastic hemiparesis’, and ‘(spastic) tetraparesis’ (2×); sensory but no motor symptoms (paraesthesia and hypesthesia) were reported in 2 patients. Two patients suffered from bladder and bowel disturbances. Three patients suffered from possible brainstem symptoms (internuclear ophthalmoplegia, dysphagia, and mild dysarthria, respectively). Of note, ataxia was present in 11/25 patients (44%) at most recent follow-up. One patient had aphasia. Auditory symptoms were present at last follow-up in 21/25 patients (84%), including latent to severe, uni- or bilateral hypoacusis and uni- or bilateral tinnitus. Scotoma was present in 18/25 patients (72%). Only one patient was free of symptoms at last follow-up.

#### Ophthalmological findings

Visual disturbances occurred at least once over the course of disease in 24/25 patients (96%; in a single patient from the ‘limited SuS’ BRAO but no clinically apparent visual disturbances were documented) and included scotoma in all cases. In 22/24 cases (92%) both eyes were affected. Fluorescein angiography was performed in 23 (96%) of the 24 patients with visual disturbances and showed BRAO in all of them (note that BRAO were an inclusion criterion for SuS group II, ‘limited SuS’; n = 5). The presence or absence of leakage was explicitly mentioned in 17 cases, and leakage was present in 16 (94%). Gass plaques [16] were present in 8/8 patients with available data.

#### Hearing impairment

Sensorineural hearing impairment occurred at least once over the course of disease in 24/25 cases (96%); the only patient without hearing impairment had bilateral tinnitus. In at least 21/23 (91%) hearing was impaired on both sides (side not documented in one case).

#### Central nervous system symptoms and magnetic resonance imaging findings

Clinical evidence of CNS involvement was present in 22/25 patients (88%) and included motor and sensory symptoms, brainstem symptoms, ataxia, bowel and bladder disturbances, neuropsychological impairment and, in one patient, aphasia. MRI revealed callosal brain lesions in 24/25 patients (96%) on-T2 weighted imaging and in 20/24 patients (83%; no data in one patient) on T1-weighted imaging. Periventricular T2 lesions were present in 24/25 patients (96%). Leptomeningeal enhancement was documented in five patients. All of the three patients without clinical signs of CNS involvement had brain MRI lesions (callosal and periventricular in two, callosal in one). Brain atrophy as detected by MRI was noted by the attending radiologist in 14/25 patients (56%).

#### Cerebrospinal fluid findings

White cell counts were mostly low (median 6.5/μl, range 0 to 42). CSF pleocytosis (≥5 cells/μl) was found in 14/22 patients at least once over the course of disease (63.6%; no lumbar puncture (LP) performed or no data available in the remaining three cases). Of the 24 documented lumbar punctures (two patients had a repeat LP), 15 (62.5%) revealed pleocytosis. The pleocytosis was generally mild (median 12 white cells/µl, range 5 to 42).

CSF protein levels were elevated in 20/25 (80%) patients. CSF-restricted oligoclonal bands (OCB) were present only in 3/23 patients (13%); repeat lumbar puncture was performed in two of the initially OCB-negative patients and was again negative for OCB. Additional, quantitative evidence for intrathecal IgG synthesis was present in two (OCB-positive) patients. Evidence for blood-CSF barrier disruption as indicated by an elevated albumin CSF/serum ratio (QAlb > Qlim(Alb) [17]) was present in seven out of nine patients (78%). Documented QAlb values ranged between 3.4 and 67.1 (median 17.5). CSF lactate levels were tested only in five patients and were normal in all of them. The CSF/serum glucose ratio was lowered in 2/4 patients (0.45 and 0.43, respectively).

#### Other laboratory findings

Auto-antibodies other than AECA were reported in 6/23 patients (26%; no data in 2), including anti-nuclear antibodies (3×), perinuclear anti-neutrophil cytoplasmic antibodies (1×), anti-thyroid peroxidase antibodies (1×), anti-thyroid microsomal antibodies (1×), and anti-C1q antibodies; in one patient, circulating immune complexes were reported. One patient each was diagnosed with IgA deficiency and activated protein C resistance, protein S deficiency, or elevated C3c. However, in none of the 25 SuS patients had a diagnosis of SLE or any other connective tissue disorder been made. AECA frequency and titers are reported below.

#### Biopsy findings

Brain biopsy was performed in two patients and showed perivenous infiltration, focal demyelination and necrosis, activated microglia, and reactive gliosis.

### Anti-endothelial cell serum antibodies

**Figure 1:**
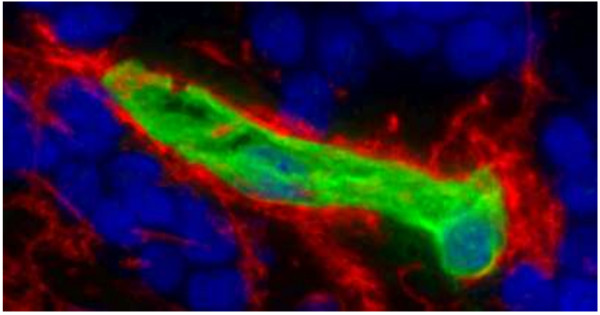
**Immunohistochemical differentiation of anti-endothelial cell antibodies (AECA) and aquaporin-4 (AQP4)-IgG by double staining with patient serum and a commercial anti-human AQP4 antibody.** While AECA (green) stain the endothelial cells, the AQP4 antibody (red) stains the astrocytic endfeet surrounding the microvasculature.

AECA were diagnosed visually based on the typical staining pattern; endothelial rather than perivascular astrocytic staining was confirmed in positive cases by using a commercial anti-AQP4 antibody as previously described [9] with none of the sera showing staining of astrocytic endfeet (Figure 1). All AECA-positive samples were negative for NMO-IgG/AQP4-Ab, the cerebellar staining pattern of which share minor similarities with that of AECA, when tested using an AQP4-specific cell based assay [13].

#### AECA frequency

**Figure 2:**
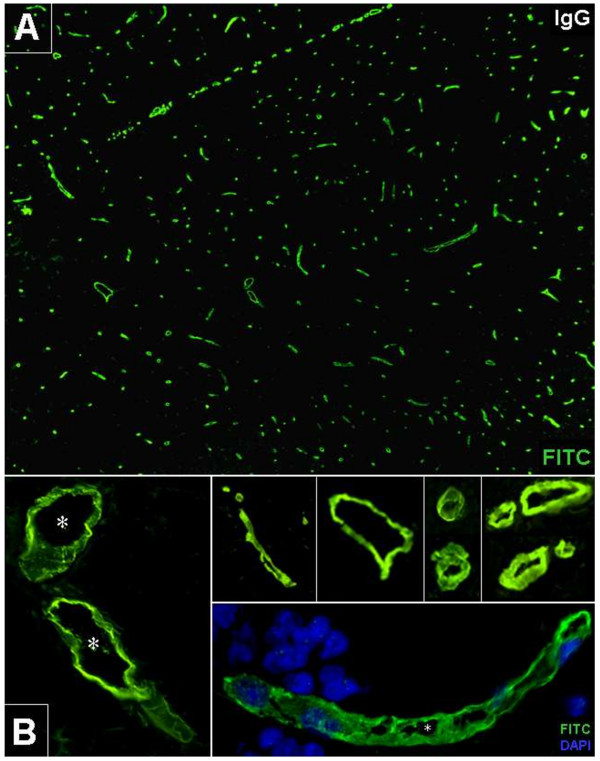
**Anti-endothelial cell IgG serum antibodies (AECA) in Susac syndrome.** A fluorescein isothiocyanate (FITC)-labeled goat anti-human IgG was used to detect binding of patient IgG to *in situ* primate brain endothelial cells (green). DAPI was used to stain cell nuclei (blue, lower right insert). Asterisks: vessel lumen. **A**: Overview at 10× magnification. **B**: Magnification at various levels (20 × to 40×).

Based on a cut-off titer of ≥1:10 (lowest dilution tested), serum AECA were detected in 7/20 patients (35%; all female) with definite SuS (group I) but in none of 5 patients with limited SuS (group II) (*P* = n.s.) (Figure 2). Follow-up samples (n = 12) were available from 7 patients; in the only one of these patients who was AECA seropositive, all four available follow-up samples were positive as well, and in the remaining 6 seronegative patients, all follow-up samples were again negative for AECA. Overall, 34.4% (11/32) of samples from patients with definite SuS were positive. In the control group, 8/70 patients (11.4%; all but one female) were positive (*P* = 0.036 versus definite SuS, if only the first SuS sample is taken into account; *P* = 0.012 versus definite SuS, if also follow-up SuS samples are taken into account); AECA were found in all three diagnostic subgroups (MS: 4/25 (16%); CTD: 2/25 (8%); and HC: 2/20 (10%)) (Table 2).

**Table 2:** Frequency of anti-endothelial cell antibodies (AECA) and AECA titers in patients with Susac syndrome (SuS) and controls according to cut-off levels

	**AECA, patients (%)**		**AECA, samples (%)**		**AECA, median titers**	**AECA, titer ranges**
	**AECA ≥1:10**					
Definite SuS	7/20 (35)	*P* = 0.036^c,d^	11/32 (34)	*P* = 0.012^c,d^	1:3200	1:100 – 1:17,500
Limited SuS	0/5 (0)		0/5 (0)		N.a.	N.a.
Controls	8/70 (11)		8/70 (11)		1:100	1:10 – 1:320
HC	2/20 (10)		2/20 (10)		1:55	1:10 – 1:100
MS	4^a^/25 (16)		4/25 (16)		1:210	1:10 – 1:320
CTD	2^b^/25 (8)		2/25 (8)		1:165	1:10 – 1:320
	**AECA >1:100**					
Definite SuS	5/20 (25)	*P* = 0.015^c,d^	9/32 (28)	*P* = 0.002^c,d^	1:3200	1:100 – 1:17,500
Limited SuS	0/5 (0)		0/5 (0)		N.a.	N.a.
Controls	3/70 (4)		3/70 (4)		1:320	1:320 – 1:320
HC	0/20 (0)		0/20 (0)		N.a.	N.a.
MS	2/25 (8)		2/25 (8)		1:320	1:320 – 1:320
CTD	1/25 (4)		1/25 (4)		1:320	1:320
	**AECA >1:320**					
Definite SuS	3/20 (15)	*P* = 0.01^c,d^	7/32 (22)	*P* < 0.0002^c,d^	1:7500	1:1000 – 1:17,500
Limited SuS	0/5 (0)		0/5 (0)		N.a.	N.a.
Controls	0/70 (0)		0/70 (0)		N.a.	N.a.
HC	0/20 (0)		0/20 (0)		N.a.	N.a.
MS	0/25 (0)		0/25 (0)		N.a.	N.a.
CTD	0/25 (0)		0/25 (0)		N.a.	N.a.

^a^3 × RRMS, 1 × PPMS. ^b^1 × SLE, 1 × Sjögren syndrome. ^c^Fisher’s exact test. ^d^Definite SuS vs controls. N.a. = not applicable.

If a more conservative cut-off of >1:100 was used, 25% (5/20) of the patients with definite SuS but only 4.3% (3/70; 2 × RRMS, 1 × SS) of the controls were positive for AECA (*P* = 0.012) and 28.1% (9/32) of the definite SuS samples (*P* = 0.002) (Table 2).

Finally, the best specificity (but the lowest sensitivity) was achieved if only titers >1:320 were considered. Titers >1:320 were absent in all of the controls (n = 70) but were found in 15% of the patients with definite SuS (n = 20; *P* = 0.010) and in 21.9% of the definite SuS samples (n = 32; *P* = 0.00018) (Table 2).

#### AECA titers

Median AECA titers were significantly higher in samples obtained from patients with definite SuS (1:3,200; range 100 to 17,500) than in samples obtained from controls (median 1:100; range 1:1:10 to 1:320) (*P* = 0.005). As stated above, none of the control samples yielded titers >1:320. See Table 2 for details.

#### IgG subclass analysis

**Figure 3:**
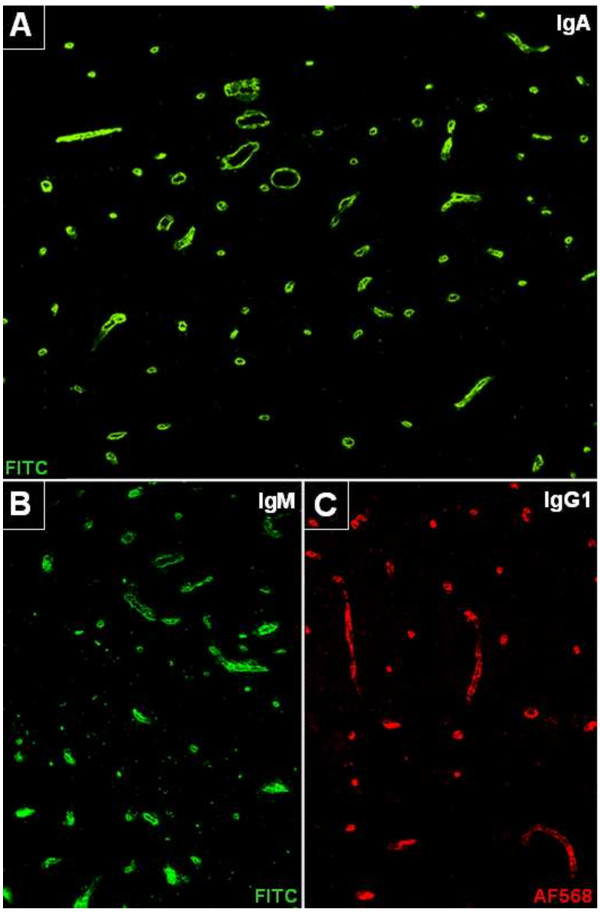
**Anti-endothelial cell antibodies (AECA) belonged to the IgG, IgA and IgM classes and included complement-activating IgG1 antibodies. A**: AECA of the IgA class. **B**: AECA of the IgM class, **C**: AECA of the IgG1 subclass. Fluorescein isothiocyanate (FITC)-labeled goat anti-human IgM or IgA antibodies (green) and a sheep anti-human IgG1 antibody (detected by means of an Alexa Fluor™568-labelled donkey anti-sheep IgG antibody; red) were used to detect binding of patient immunoglobulin to *in situ* primate brain endothelial cells. See Results section for details.

IgG subclass analysis was performed in the five highly AECA-positive SuS serum samples and revealed antibodies of the strongly complement-activating IgG1 and IgG3 subclasses in all cases (Figure 3C) but not in five randomly chosen control samples; additional AECA of the IgG2 and the IgG4 subclasses were present in 5/5 and 4/5 samples, respectively, when tested at a dilution of 1:32. Titration of serum samples from three total IgG-AECA-positive patients revealed IgG1-AECA titers of >1:10,000 (total-IgG-AECA: 1:17,500), 1:1,000 (total-IgG-AECA: 1:3,200), and 1:1,000 (total IgG-AECA: 1:1,000), respectively (note as a caveat that total-IgG titers and IgG1 titers are not directly comparable, since different detection antibodies are used).

#### Anti-endothelial IgM and IgA antibodies

All IgG-AECA-positive SuS samples (n = 11) were in addition tested for AECA of the IgA and the IgM class. A total of 10 of these samples (90.9%), were positive for IgA-AECA and 5 (45.5%) for IgM-AECA (Figure 3A, B).

#### Effect of disease activity

No association between AECA seropositivity or AECA titers and acute disease was found in the present cohort: While four AECA-positive SuS samples were obtained during phases of active disease, nine AECA-positive SuS samples (including the four samples with the highest AECA titers (1:17,500, 1:10,000, 1:10,000, 1:7,500)) were taken during remission.

#### Effect of disease duration

Median disease duration in the AECA-positive samples was 2 years with a range of 0 to 17 years. Although a positive correlation of AECA titers with time since onset in years was found (*P* = 0.002), this may simply reflect the inclusion of five samples from a single patient with long disease duration that were taken over a relatively short period of time (29 months) and were all highly positive for AECA. Moreover, it is generally difficult to define the exact time of SuS onset, since the condition can start with non-specific neuropsychological symptoms.

#### Treatment effects

To evaluate whether AECA seronegativity was due to immunotherapy in some patients, we analyzed the patients’ treatment status at the time of blood sampling and within 12 months before blood sampling. However, the majority of AECA-negative samples was taken during IS/IM-free intervals (Table 3, row 1 to 2). Moreover, all 15 AECA-negative untreated samples were obtained >12 months after the last administration of an IS/IM treatment (including steroids) or had never been treated with IS or IM drugs. On the other hand, a more detailed analysis distinguishing between the various IS/IM treatments used (Table 3, row 3 to 8) showed that all samples obtained during treatment with either azathioprine or cyclophosphamide (n = 5) were negative for AECA. The frequency of AECA was also lower among samples taken during steroid treatment (3 × positive, 8 × negative), though co-treatments with other IS/IM could have played a role as well in the AECA-negative steroid-treated patients (3 × cyclophosphamide, 2 × IVIG, 2 × AZA). Among the AECA-positive samples, six were obtained during periods of active treatment with IS and/or IM drugs and four during treatment-free intervals (Table 3, rows 1 to 2).

**Table 3:** **Immunosuppressive and immunomodulatory treatments at the time of blood sampling (N = 37) in patients (N = 25) with Susac syndrome (SuS), stratified according to anti-endothelial cell antibodies** (**AECA) serostatus**

	**SuS samples, total**	**SuS samples, AECA-positive**	**SuS samples, AECA-negative**
Untreated	19	4	15
Treated^a^	16	6	10
Steroids	11	3	8
IVIG	6	3	4
Azathioprine	2	0	2
Cyclophosphamide	3	0	3
Mycophenolate mofetil	3	2	1
Methotrexate	1	1	0
Unknown	2	1	1

^a^Note that the values in rows 3 to 8 of each column do not sum up to the values given in row 2 of the same column, since some patients received more than immunosuppressive/immunomodulatory treatment at the time of blood sampling. AECA, anti-endothelial cell antibodies; IVIG, intravenous immunoglobulins; SuS, Susac syndrome.

#### Clinical and paraclinical findings in seropositive patients

No significant clinical and paraclinical differences were observed between AECA-positive and AECA-negative patients (not shown). Visual disturbances were bilateral and associated with the presence of scotomata in 6/6 seropositive patients (no exact data in one); hearing loss was bilateral in 6/7. Encephalopathic signs and symptoms included fatigue, cognitive/mnestic impairment, paresis, spasticity, hypaesthesia, and ataxia. Gass plaques were present in the single AECA-positive patient with available data. None of the AECA-positive patients was positive for OCBs (0/7; confirmed by a repeat LP in one) or had quantitative evidence of intrathecal IgG synthesis as indicated by an increased IgG CSF/serum ratio (0/6; no data in one). QAlb was documented in a single AECA-positive patient and was elevated, suggesting possible BBB damage. Three (43%) AECA-positive patients had a history of mild CSF pleocytosis (11 to 42 cells/μl).

#### Comparison with rodent tissue sections

AECA titers in the SuS group were much lower when tested on rodent tissue in all cases (for example, sample #1: 1:10,000 on primate tissue versus 1:32 on rat tissue versus negative on mouse tissue; sample #2: 1:7,500 versus 1:100 versus 1:32, sample #3: 1:1,000 versus 1:1,000 versus 1:32). Moreover, using rodent tissue sections, 11 additional control samples came up positive (titer range 1:10 to 1:320), resulting in lower specificity.

## Discussion

This is one of the largest series of patients with SuS so far. It is a potential advantage that our data are derived from a single series with uniform inclusion criteria. By contrast, almost all of our previous knowledge on SuS was based on reports of single cases and small case series, only a few of which included more than four patients. The completeness of data provided in those reports, some of which were published only in abstract form, varied widely, as did the inclusion criteria for patients with limited SuS. We therefore applied stricter criteria for the inclusion of patients with abortive SuS, all of whom had to present with BRAO, and aimed at collecting a more comprehensive and complete set of data than in some of the previous reports.

Our study reveals a broad spectrum of neurological signs and symptoms associated with SuS, including, in addition to headache, motor, sensory, brainstem and cerebellar symptoms, aphasia, and cognitive and memory decline. In addition, all patients with SuS had periventricular and/or callosal brain lesions on MRI. These findings, the mostly relapsing course, and the occasional presence of CSF-restricted OCB underlines the relevance of SuS as a rare differential diagnosis of MS, especially if disease starts with isolated encephalopathic symptoms, which was the case in 60% of our patients. The 7-T MRI and optical coherence tomography have recently been shown to be of help in the differential diagnosis of MS and SuS [6,18].

The findings that visual and hearing impairments were bilateral in 92% and 96% of cases, respectively, that 64% of the patients had neurological symptoms at last follow-up and, importantly, that more than half of them exhibited brain atrophy, despite both immunosuppressive and antithrombotic treatments having been tried in most cases, characterize SuS as a severe disease.

Importantly, from both the pathogenetic and the diagnostic point of view, around 30% of patients with definite SuS were positive for serum AECA. The fact that AECA were present at high titers (up to 1:17,500) in some of these patients and were found to belong to the complement-activating IgG1 and Ig3 subclass in all of them, suggests that AECA might possibly play a role in a subset of patients with SuS. IgM antibodies, which are considered even stronger complement activators, were present in around half of the cases. Of note, AECA were present in all five samples obtained from a single patient with SuS taken over a period of 29 months, indicating that AECA seropositivity was not an accidental or transitory finding.

A possible role for AECA in some patients with SuS is further supported by the finding that median AECA titers were significantly higher in the SuS group than in the control group. AECA titers of >1:320 were exclusively found in patients with SuS; by contrast, they were demonstrated in none of the 70 controls, including 25 patients with CTD. Of special note, frequency and titers of AECA were higher in the SuS group than in the control group despite a higher rate of immunosuppressive treatment at the time of blood sampling in the SuS group (neither the HC nor the MS patients had ever received long-term IS treatment).

As AECA seronegativity was not strictly associated with IS/IM treatment or remission, the lack of AECA seropositivity in some patients could indicate that

1. SuS is an etiologically heterogeneous syndrome, which is not caused by AECA in all cases; and/or

2. AECA are an optional secondary phenomenon following endothelial damage of other origin (for example, T cell mediated) not occurring in all patients.

In fact, a broad range of conditions are capable of causing microinfarctions and/or BRAO, and etiological heterogeneity has been demonstrated over the last few years in a number of autoimmune CNS syndromes, such as limbic encephalitis, subacute cerebellar degeneration, neuromyelitis optica (NMO) and myasthenia gravis [19-22]. Importantly, AECA were absent also in a number of patients with active disease, demonstrating that the presence of AECA, at least in some cases, is not a *conditio sine qua non* for SuS flares.

Interestingly, almost 80% of all patients with available data showed an elevated CSF/serum albumin ratio, which in the absence of substantial CSF flow alterations (as seen, for example, in spinal canal stenosis) is thought to mainly reflect blood–brain barrier dysfunction. This is well in line with results from histopathological and ophthalmoscopic (fluorescein dye leakage, arterial wall plaques) studies showing vascular damage in SuS.

The absence of CSF-restricted OCB in most patients does not *per se* argue against SuS being an autoimmune CNS disorder and against AECA being involved in the pathogenesis of CNS damage, since the antibodies’ antigen might well be on the luminal side of the endothelium and thus directly accessible to AECA in the peripheral blood. Similarly, autoantibodies to AQP4 in NMO are mainly produced in the periphery [23-25]. OCBs were found to be frequently missing also in other CNS disorders of putative autoimmune etiology [26-28].

Since publication of our index case [9], a single study on AECA in SuS has been published, the authors of which strongly advocated a role of such antibodies in the pathogenesis of the syndrome [10]. However, it is unknown whether the antibodies detected in that study were indeed AECA, whether they bound to brain endothelial antigens, and whether they were specific for SuS, since only endothelial cells (EC) but no control cells (as naturally included in the tissue sections used in the present study) were used in some of the experiments, cutaneous EC instead of brain EC were employed, no control sera were used in the IHC experiments, statistical significance levels were provided only for a single assay, and the histopathological data demonstrating endothelial pathology were derived from the only of all patients that had not been tested for AECA. Moreover, patient numbers were relatively low. Finally, only 5 of the 11 patients tested for AECA had the typical triad of SuS, and MRI data, which could have supported the diagnosis especially in the ‘limited SuS’ cases, were not supplied.

Unless the exact role of AECA in the pathogenesis of SuS has been better defined, we believe that the detection of AECA alone, especially if present only at low titer, does *per se* not justify B cell or antibody targeted treatments. However, an effect of IS/IM treatment at least in a subset of patients with SuS is suggested by a number of anecdotal reports and small retrospective case series. It is recommended that IS/IM treatment of patients with SuS should be performed in the context of controlled clinical trials or, at least, treatment registers in the future. Such trials or registers should include standardized serum testing for AECA as a prerequisite for investigating the role of AECA as well as the effect of IS/IM treatment on AECA serostatus and titers in SuS in a more definite way.

For routine autoantibody testing, many clinical laboratories use mouse or rat tissue instead of primate tissue. While rodent tissue sections are more easily available, the use of non-primate tissue may be problematic due to interspecies differences in antigen structure. To evaluate whether primate tissue can be replaced by rodent tissue, all SuS samples seropositive on primate tissue were tested in addition on mouse and rat cerebellum tissue sections. Based on that direct comparison, we recommend that future studies on AECA in SuS using IIF should use primate tissue, which yielded higher sensitivity, titers and specificity than rodent tissues in the present study.

It is a potential limitation of our study that some patients were treated with IS/IM drugs at the time of blood sampling and that some samples were taken during periods of clinical remission. However, as mentioned above, AECA were negative also in a relevant number of samples taken from untreated patients or taken during active disease. Moreover, several of our AECA-positive patients had very high titers also during remission (similarly, autoantibodies remain detectable during remission in many neurological and non-neurological autoimmune disorders such as AQP4-Ab-positive NMO [15,29], myasthenia gravis [30] or SLE [31]. These findings suggest that treatment and disease activity are not the only factor determining AECA serostatus; instead, AECA may be truly absent in some cases of SuS as discussed above.

In summary, our study provides systematic clinical and paraclinical information derived from a single large series of patients with SuS. In addition, we demonstrate that complement-activating IgG1 and IgM AECA are present in a subset of patients with definite SuS, in some cases at high level. Future studies are now warranted to evaluate the exact pathogenic impact of AECA in SuS and to identify their antigenic target.

## Abbreviations

AECA: anti-endothelial cell antibodies; BRAO: branch retinal artery occlusions; CNS: central nervous system; CTD: connective tissue disorders; EC: endothelial cells; FITC: fluorescein isothiocyanate; IFA: immunofluorescence assay; IHC: immunohistochemistry; IM: immunomodulatory; IS: immunosuppressive; IVIG: intravenous immunoglobulins; LP: lumbar puncture; MRI: magnetic resonance imaging; MS: multiple sclerosis; NMO: neuromyelitis optica; OCB: oligoclonal bands; SLE: systemic lupus erythematosus; SuS: Susac syndrome.

## Competing interests

KF and WS are employees of Euroimmun AG, Lübeck. Euroimmun kindy provided the tissue sections used in this study. The other authors have no competing interests.

## Authors’ contributions

SJ, IK, JD, FP and BW conceived the study. SJ designed the study, performed the experiments, analyzed the data, and wrote the manuscript. SJ, IK, JD, JS-G, ZI, EE, CC, MR, OA, XM, EBR, FP and BW were involved in patient care and retrospective data analysis. KF and WS were involved in antibody testing. All authors were involved in critically revising the manuscript for important intellectual content. All authors read and approved the final manuscript.

## Authors’ information

FP and BW are equally contributing senior authors.
